# Improving the Heat and Ablation Resistance of Silicone Rubber Composites by Incorporating Hollow Microspheres

**DOI:** 10.3390/polym14183846

**Published:** 2022-09-14

**Authors:** Jinfeng Tian, Liwei Yan, Hao Zhang, Shengtai Zhou, Shuang Xia, Huawei Zou

**Affiliations:** The State Key Laboratory of Polymer Materials Engineering, Polymer Research Institute of Sichuan University, Chengdu 610065, China

**Keywords:** ablation resistance, hollow microspheres, silicone rubber, thermal conductivity

## Abstract

For thermal protection materials (TPMs) which are used to protect space vehicles from extreme thermomechanical environments, the thermal conductivity of the original material and the char layer that has formed during ablation plays a significant role in determining the ablation performance. In order to investigate this, in this study, we introduced glass hollow microspheres (GHMs), phenolic hollow microspheres (PHMs), and acrylonitrile-methyl methacrylate copolymer hollow microspheres (AMHMs) into silicone rubber (SR), and the ablation performance of these composites was systematically studied. The thermogravimetric results showed that the residue yield of the SR composites was increased with the incorporation of the hollow microspheres. Compared to the SR composites without the hollow microspheres, the residue weight values under 800 °C (R_800_) of the SR composites with the 30 parts of fumed silica per hundred of the SR (phr) addition of GHMs, PHMs, and AMHMs were increased from 10.11% to 21.70%, 18.31%, and 20.83%, respectively. The ablation tests showed that the addition of the AMHMs enhanced the ablation performance of the SR composites because the linear ablation rates and the backplane temperature were clearly decreased when compared to the SR composites without the hollow microspheres. This work provides an effective and potential method for preparing thermal protection materials with an improved ablation performance.

## 1. Introduction

Thermal protection materials (TPMs) play a critical role in the aerospace industry and are used to protect space vehicles from extreme thermomechanical environments [1,2,3,4]. Polymeric ablative materials (PAMs) are the most widely used materials for thermal protection due to their good designability, processability, and low density. PAMs are divided into rigid ablative materials (RAMs) and flexible ablative materials (FAMs) according to the elongation and flexural properties of the polymer matrix. Generally, RAMs refer to PAMs with a hard resin as a matrix [5,6,7,8], whereas FAMs refer to those with a rubber elastomer as the matrix [9,10,11,12,13]. With the rapid development of aerospace technology, more and more dynamic components are designed to complete deformation during the high-speed flight process. In contrast to the conventional RAMs, which are constrained by their high-temperature brittleness and low-deformation capability to meet the deformation requirements [14], the FAMs have a large elongation-at-break and certain flexural properties, which enable them to accommodate large-load deformation during high-speed flight [9,15,16]. Therefore, FAMs are very attractive candidates for the thermal protection and sealing of dynamic structures, as well as for large deformation and thermal stress matching.

To date, the most widely used flexible PAMs are usually based on nitrile rubber (NR) [17], ethylene propylene diene monomer (EPDM) [9,11], polyphosphazene elastomers [10,18], and silicone rubber (SR) [12,13,19,20], etc. It is worth noting that the SRs with the Si-O-Si backbone possess unique organic/inorganic properties, exhibiting excellent oxidation resistance and heat resistance [21], which makes them the most promising flexible matrix for FAMs in an oxidative ablation environment. In general, pure SR cannot form a dense char layer during ablation, which makes it difficult to apply directly in aerospace [20,22]. Therefore, fiber fillers [23,24], powder fillers [19,25,26], and nano-fillers [27,28], etc., are added to SR to improve its ablative properties. Large filler additions, however, tend to increase material density, which can result in a decrease in the payload of vehicles. Additionally, these fillers tend to improve the thermal conductivity of the original materials [29] and the char layer [30], causing heat from the outside to be conducted by the inner material during ablation and therefore accelerating the pyrolysis of the SR matrix. Research has shown that the thermal conductivity of the material inherent and the char layer formed during its ablation is a key factor in determining the thermal insulation performance of TPMs. The low thermal conductivity and the density of the material could lead to the excellent thermal insulation performance of TPMs. Due to the special hollow-core structure, the hollow microspheres show low density and heat insulation characteristics and they are commonly used to reduce the density of FAMs [31,32]. However, there are few reports on the SR-based FAMs which have been modified by low-density hollow microspheres.

In this paper, PBO fibers were used to enhance the char layer, and glass hollow microspheres (GHMs), phenolic hollow microspheres (PHMs), and acrylonitrile-methyl methacrylate copolymer hollow microspheres (AMHMs) were introduced into the SR composites to prepare the ablative SRs. The inorganic GHMs, the organic PHMs with high residue under high temperature, and thermoplastic AMHMs show different heat resistance properties. In contrast to previous research, the present study investigated the influence of hollow microspheres with different heat resistance on the ablation resistance of SR composites. The effects of various hollow microspheres on the ablation behavior of the SR composites were compared in detail. The ablation thermal insulation properties of the materials were examined utilizing ablation tests and backplane temperature measurements. The microstructures for the char layers of different materials after ablation were analyzed by scanning electron microscopy (SEM). 

## 2. Experimental Section

### 2.1. Materials

**Figure 1:**
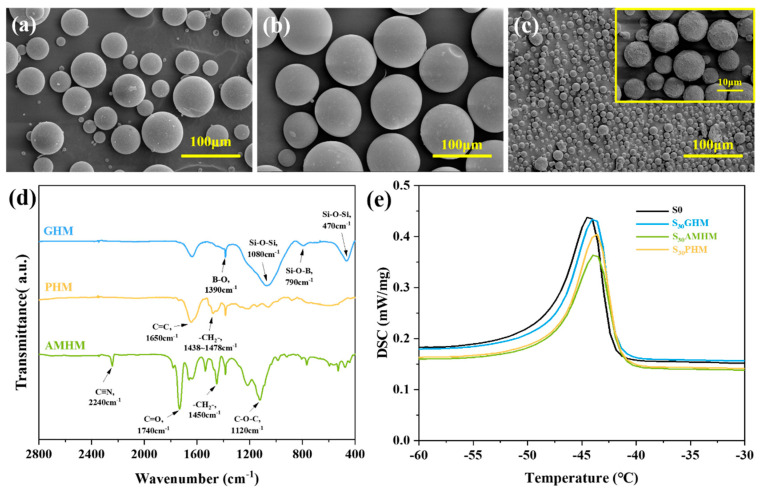
(**a**–**c**) SEM images of GHMs, PHMs, and AMHMs; (**d**) FTIR spectrum of GHMs, PHMs, and AMHMs; (**e**) DSC curves of S0, S_30_GHM, S_30_AMHM, and S_30_PHM.

**Figure 2:**
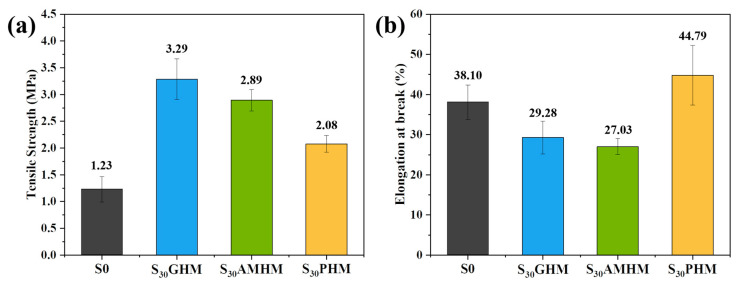
(**a**) Tensile strength and (**b**) elongation-at-break of S0, S_30_GHM, S_30_PHM, and S_30_AMHM.

The epoxy-modified liquid silicone rubber (E-LSR) was prepared in our laboratory as per [32]. The tetraisopropyl titanate (TPT) was purchased from J&K Scientific Co., Ltd. (Beijing, China). The fumed silica (AEROSIL 200) was supplied by Evonik Degussa (Essen, Germany). The curing agent and the poly (p-phenylene-2, 6-benzoxazole) (PBO) fibers with a length of 3 mm and a diameter of 12.5 μm were provided by China BlueStar Chengrand Research Institute of chemical Industry (Chengdu, China). The glass hollow microspheres (GHMs) were purchased from Beijing Forsman Scientific Co., Ltd. (Beijing, China). The acrylonitrile-methyl methacrylate copolymer hollow microspheres (AMHMs) were obtained from Akzo Nobel N.V (Amsterdam, The Netherlands). The phenolic hollow microspheres (PHMs) were provided by the Institute of Chemistry Chinese Academy of Sciences. Firstly, the phenol-formaldehyde resol resin, the pre-treated foaming agent, and the surfactant were mixed by ultrasonication. Then, the mixture was added dropwise to a hot paraffin oil bath, which was continually stirred until it foamed and cured. Finally, the collected microspheres were washed by ultrasonication and dried to obtain hollow microspheres. The microstructure and the Fourier transform infrared (FTIR) spectrum of hollow microspheres are shown in Figure 1 and Figure 2, respectively.

### 2.2. Sample Preparation

Firstly, 5 parts of fumed silica per hundred of the E-LSR (phr) was gradually added into the E-LSR and mixed with mechanical stirring until uniform. This mixture was then processed with a three-roller mill to make the silica uniformly dispersed. Then, the PBO fibers and the hollow microspheres were gradually added to the above mixture, then mixed within a chamber mixer. The corresponding content of the curing agent and the catalyst were added and mixed for 2~3 min. The mixture was placed into the mold and vulcanized in the flat-plate vulcanizer for 24 h (room temperature at 10 MPa). Finally, the mixture was kept at room temperature for a week to make it completely cured, and then the samples were cut for use. The final specimens of the SR composites with the addition of the different hollow microspheres were named S_N_GHM, S_N_PHM, and S_N_AMHM, where N means the addition of the corresponding hollow microspheres. The formulation of different samples is listed in Table 1. Comparisons were made with pure SR and SR composites prepared without hollow microspheres (S0).

### 2.3. Characterization

Fourier transform infrared spectroscopy was carried out using a Nicolet 570 infrared spectrophotometer (Nicolet 570, Nicolet, Green Bay, WI, USA) with a scan range of 4000–400 cm^−1^. Differential scanning calorimetry (DSC) measurements were conducted using a differential scanning calorimeter (DSC 214 Polyma, NETZSCH, Selb, Germany). About 4 mg of the samples was placed in an aluminum crucible and heated at a heating rate of 5 °C/min from −70 °C to 30 °C. 

The tensile strength and the elongation-at-break of S0, S_30_GHMs, S_30_PHMs, and S_30_AMHMs were measured by an Instron universal testing instrument (Instron 5567, Instron, Norwood, MA, USA) at a stretching rate of 500 mm/min according to ISO 37:2011. All results for each sample were the average of 4 specimens.

Thermogravimetric analysis (TGA) was performed on a thermogravimeter (TGA 209F1, NETZSCH, Selb, Germany). About 2~10 mg of the samples were heated from room temperature to 800 °C at a heating rate of 10° C/min under a N_2_ atmosphere. The thermal conductivity measurements of the samples were carried out by a thermal constant analyzer (TPS 2500, Hot Disk, Göteborg, Sweden).

The ablative properties of the samples were evaluated by an oxy-acetylene torch. The samples with dimensions of 40 × 40 × 10 mm^3^ were exposed to a steady heat flux of 2 MW/m^2^ for 30 s. The pressure of oxygen (O_2_) and acetylene (C_2_H_2_) were 0.4 and 0.095 MPa, respectively, with gas flow rates of 10,350 and 9500 mL/min, respectively. The torch nozzle with an inner diameter of 2 mm was placed vertically on the tested samples, and the distance between the tip and the sample was 10 mm. The backplane temperature during the ablation test was recorded by K-type thermocouples. After ablation, the samples were cooled to room temperature, and the char layers were peeled off. The change in thickness of the specimens before and after ablation was measured, and the linear ablation rate was obtained based on the following formula:

(1) $$Rd=\frac{\Delta d}{t}=\frac{d1-d2}{t}$$

where *R_d_* represents the linear ablation rate (mm/s); *d*_1_ and *d*_2_ are the original thickness of the specimen before ablation and the thickness of the ablated specimen after removing the char layer (mm), respectively; and *t* means the testing time.

Scanning electron microscopy (SEM; Apreo S HiVoc, Thermo Fisher Scientific, Mundelein, IL, USA) with an acceleration voltage of 5 kV was employed to observe the microstructure of the char layers after ablation.

## 3. Results and Discussion

### 3.1. Structure Analysis of the Microspheres and the Corresponding Composites

As is shown in Figure 1a–c, the GHMs, the PHMs, and the AMHMs all have a spherical shape with diameters of 55–100 μm, 45–100 μm, and 9–15 μm, respectively. The FTIR was performed to analyze the chemical structure of the three different hollow microspheres and the results are presented in Figure 1d. For the GHMs with the major component of borosilicate, the bands at 1390 cm^−1^ and 790 cm^−1^ correspond to the stretching vibration of B-O and Si-O-B, respectively. The broad band at 1080 cm^−1^ and 470 cm^−1^ is ascribed to the existence of the stretching and bending vibrations of Si-O-Si, respectively. In the spectra of the PHMs with phenolic resin shells, the C=C stretching vibration at 1650 cm^−1^ and the C-H bending vibration at 1438~1478 cm^−1^ indicated the presence of a benzene structure. In the spectra of AMHMs, the peak corresponding to 2240 cm^−1^ showed the stretching vibration of C≡N related to acrylonitrile units, and the peaks at 1740 cm^−1^ and 1120 cm^−1^ are related to the stretching vibration of the ester groups on the methyl methacrylate units.

The DSC tests were conducted to evaluate interfacial interaction between the hollow microspheres and the SR matrix by analyzing the melting behavior of the SR composites. The DSC curves of the S0, S_30_GHM, S_30_AMHM, and S_30_PHM are shown in Figure 1e and Table 2. The melting point (T_m_) reflects the interaction between the hollow microspheres and the SR matrix. As the interaction between microsphere increases, the mobility of molecular chains of the SR matrix is restrained so that the T_m_ increases. It can be concluded that the S_30_PHM has the biggest increase in T_m_, as the interfacial interaction of S_30_PHM is the best. Microspheres forming fine interfacial interactions with the SR matrix may contribute to the carbonization under high temperature and the improvement of ablation resistance.

### 3.2. Mechanical Properties

The results for tensile strength and elongation-at-break of the S0, S_30_GHM, S_30_PHM, and S_30_AMHM specimens are shown in Figure 2a,b, respectively. The mechanical strength factors of the SR composites were complex and diverse, including interfacial compatibility, particle size, and dispersion. The low contact area of fillers may have enhanced the physical or chemical interaction between the fillers and the SR matrix so that the SR composites with the addition of hollow microspheres had a stronger tensile strength. The introduction of the hollow microspheres caused the reduction in the degree of crosslinking, which is in turn attributed to the increase in the elongation-at-break; however, the elongation-at-break always decreased due to the restraining motion of the SR molecular chains by the microspheres. 

### 3.3. Thermogravimetric Analysis of Different Hollow Microspheres

**Figure 3:**
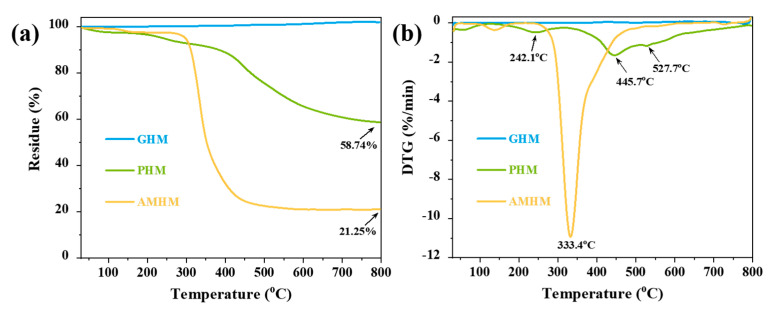
(**a**) TG and (**b**) DTG curves of three types of hollow microspheres in a N_2_ atmosphere.

The thermogravimetric (TG) and the derivative thermogravimetric (DTG) curves of the three types of low-density hollow microspheres in a N_2_ atmosphere are shown in Figure 3. The initial decomposition temperature (T_onset_, calculated by 5% mass loss), the maximum degradation-rate temperature (T_max_), and the residue weight under 800 °C (R_800_) of each microsphere are listed in Table S1. The GHMs exhibited exceptional heat resistance with no mass loss throughout the heating process, since their major component, borosilicate, has a melting point of up to 1500 °C. The PHMs showed an obvious weight loss with an R_800_ of 58.74%. During the degradation process, the PHMs showed three degradation stages with a T_max_ of 242.1 °C, 445.7 °C, and 527.7 °C, respectively (Figure 3b). These three stages corresponded to (i) the removal of end groups such as hydroxymethyl and carbonyl; (ii) the cleavage of methylene bridges; and (iii) the dehydration and cyclization of phenolic hydroxyl groups, respectively [33,34]. The AMHMs experienced a violent decomposition rate with an R_800_ of 21.25% at 800 °C. The weight loss of the AMHMs occurred mainly between approximately 279.0 and 464.0 °C with the weight loss peak registering at 333.4 °C. This weight loss was mainly due to the degradation of the thermoplastic resin shell and the release of internal alkane gases. In short, the thermal stability of the hollow microspheres demonstrated the following order: GHM > PHM > AMHM.

### 3.4. Thermogravimetric Analysis of the SR Composites with Different the Contents of Hollow Microspheres

**Figure 4:**
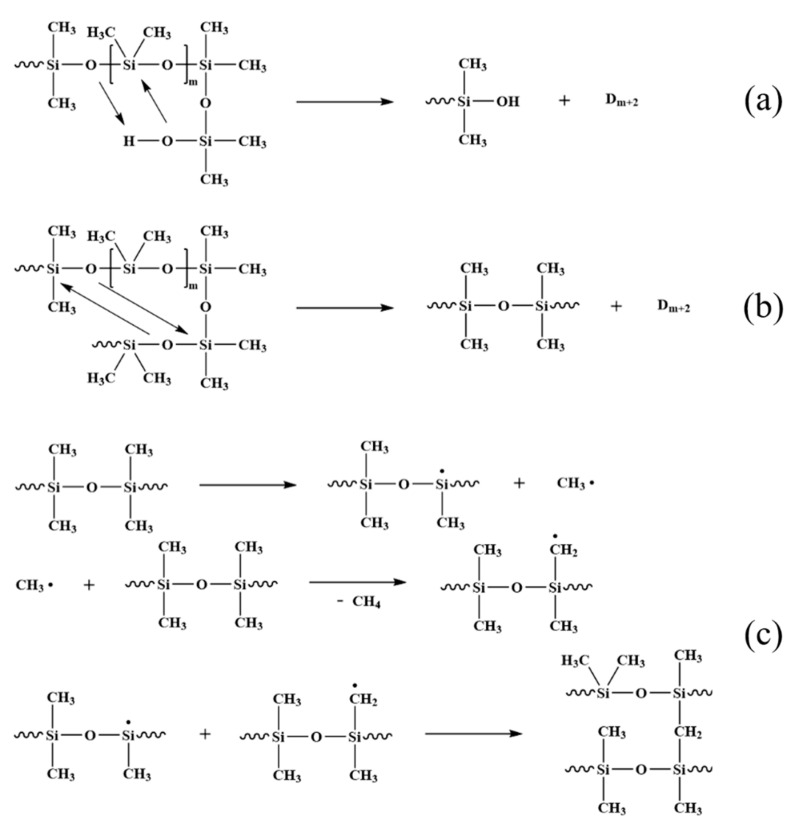
The degradation mechanism of the SR by (**a**) a zipper-type (back-bite) degradation [35,36] and (**b**) a random scission [36], as well as (**c**) a radical mechanism at high temperature [37,38].

The degradation mechanism of the SR during heat exposure is demonstrated in Figure 4. A zipper-type (back-bite) degradation and a random scission are the primary thermal degradation mechanisms of silicone rubber molecular chains [35,36]. A random scission refers to the removal of a stable ring structure formed by the intra- or intermolecular chain arrangement of the molecular chain of silicone rubber. In general, the vinyl-capped polysiloxanes primarily degrade via a random fracture, whereas the hydroxyl-capped polysiloxane primarily break down via a zipper-type back-bite process, as well as a random scission at higher temperatures. In addition, at higher temperatures, the polysiloxane molecular chains undergo degradation reactions in a radical mechanism, resulting in the release of oligomers and methane [37,38]. This process generates macromolecular chain radicals that combine with each other to form a highly cross-linked structure and further provides the conditions for the formation of the Si-C-O ceramic structures. The radical degradation mechanism occupies a large proportion of the ablation process.

**Figure 5:**
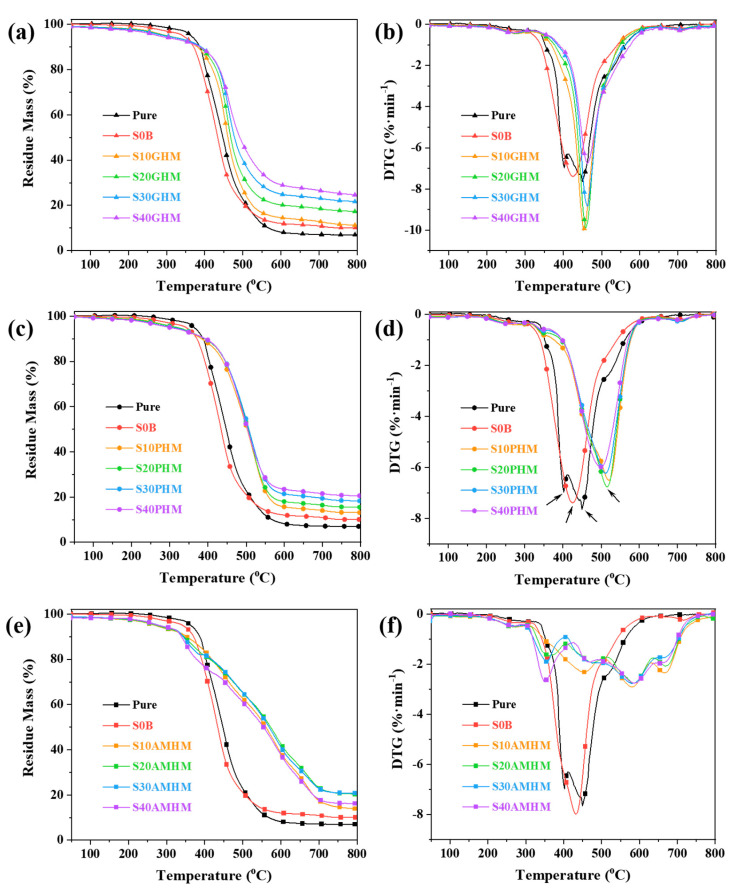
TG and DTG curves of the SR composites containing different contents of hollow microspheres in N_2_ atmosphere: (**a**,**b**) S_N_GHMs, (**c**,**d**) S_N_PHMs and (**e**,**f**) S_N_AMHMs.

Figure 5 exhibits the TG-DTG curves of the SR composites containing the different microspheres in a N_2_ atmosphere. As illustrated in Figure 5a,b, the T_onset_ of the S_N_GHM steadily declined as the addition of the GHMs increased. This is because the hydroxyl groups on the surface of the GHMs may have accelerated the unzipping degradation of the siloxane molecular chains [39]. However, the T_max_ of the SR composites was postponed with the addition of the GHMs. This could be attributed to the fact that the presence of the GHMs restricted the movement of the molecular chains and inhibited the unzipping decomposition. In addition, the residue mass of the SR composites at 800 °C (R_800_) gradually increased with the increasing content of the GHMs. To determine whether the introduction of the GHMs affects the thermal degradation process of the SR, the theoretical residual mass (R_T800_) was then calculated, under the assumption that the degradation processes of the GHMs and the SR matrix do not interfere with each other. The T_onset_, T_max_, R_800,_ and R_T800_ results are shown in Table S2. These results show that the actual residual weight is generally lower than the theoretical value, indicating that the GHMs aggravated the mass loss of the SR composite, although they delayed its maximum thermal degradation rate temperature. This is probably due to the GHMs restricting the combination of the SR molecular chain radicals and inhibiting the formation of the three-dimensional Si-C-O structure, while the escape of the small molecular gases produced by pyrolysis resulted in a decrease in the residual mass.

Figure 5c,d show the TG and DTG curves of the S_N_PHMs in a N_2_ atmosphere. The T_onset_ of the S_N_PHMs decreased with the increasing PHM content. This was because the hydroxyl groups on the surface of the PHMs catalyzed the unzipping degradation of the siloxane molecular chains. The T_max_ of the SR composites was delayed with the introduction of the PHMs and this is due to the fact that the PHMs distributed in the matrix hindered the movement of the siloxane molecular chains and inhibited its bite-back degradation. However, the delay in the T_max_ of the SR composites reduced with the increase in additional PHMs. When the SR matrix degraded, the constraint of the matrix on the PHMs was also weakened, so that the PHMs underwent a violent thermal degradation, which was not beneficial for the delay in the T_max_. The R_800_ of the S_N_PHMs at 800 °C gradually increased as the addition of the PHMs increased, but its R_800_ was always slightly lower than the corresponding R_T800_, indicating that although the PHMs could delay the T_max_ of the SR, this would not be attributed to the char yield. This is probably because the PHMs inhibited the formation of a three-dimensional cross-linked network, while the small molecule and the cyclic oligomers escaped with the N_2_ flow. 

As can be seen in Figure 5e,f, the T_onset_ of the SR composite shifted to a lower temperature after the incorporation of the AMHMs. This was attributed to the poor thermal stability of the AMHMs. Moreover, the thermal degradation stages of the SR composite containing the AMHMs increased from one to four in comparison to the control sample without the AMHMs, and the maximum thermal degradation of these four degradation stages was relatively small. In addition, all the R_800_ measurements of the SR composites with the AMHMs were considerably higher than their R_T800_ measurements, demonstrating that the AMHMs changed the thermal degradation process of the SR. Specifically, the AMHMs not only delayed the degradation of SR, but also significantly reduced the weight loss in each degradation stage, and finally dramatically increased the residual mass of the SR composites. Therefore, the AMHMs significantly improved the heat resistance of the silicone rubber.

### 3.5. Ablation Properties

**Figure 6:**
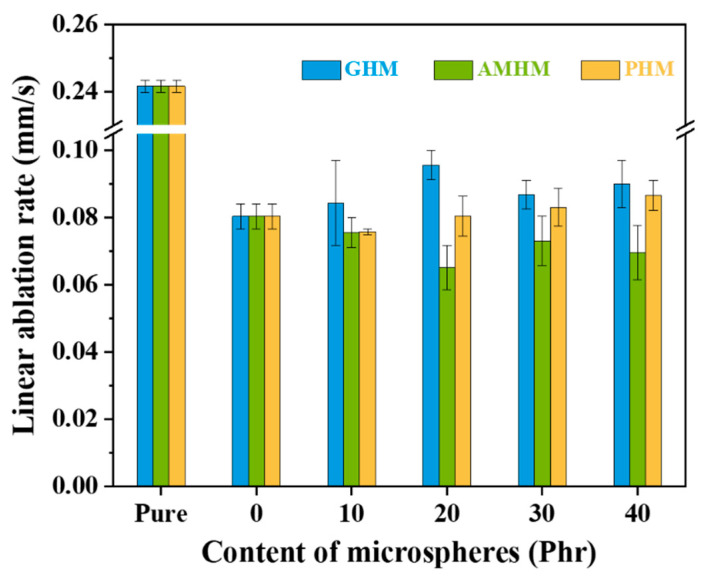
The linear ablation rate of silicone rubber composites containing three types of hollow microspheres with different loading.

Figure 6 shows the linear ablation rates of the S_N_GHM, S_N_PHM, and S_N_AMHM. The corresponding results are shown in Table S4. For the S_N_GHM, when the addition of the GHMs was less than 20 phr, the linear ablation rate of the S_N_GHMs increased with the increasing GHMs content. Under a temperature of about 3000 °C, softening or melting of the GHMs could significantly increase the thermal conductivity of the char layer. Therefore, the external heat conduction of the material would accelerate the decomposition of the SR matrix. In addition, the hydroxyl groups on the surface of the GHMs could catalyze the thermal degradation of the SR matrix, which would be harmful to the formation of a stable char layer. 

With the increasing PHMs, the linear ablation rate of the S_N_PHM gradually improved. This could be due to the fact that during the ablation, the PHMs could transform into almost-intact carbon microspheres due to their high char yield, therefore enhancing the thermal conductivity of the char layer. Thus, the external heat could not be effectively insulated and the thermal destruction of the internal material would be accelerated. The addition of the AMHMs reduced the linear ablation rate of the S_N_AMHM composites. Under a high temperature, due to the decomposition of the thermoplastic resin shell of the AMHMs, the release of the pyrolysis gas and an internal alkane could facilitate the formation of a rich-porous char layer with a low thermal conductivity. In addition, the AMHMs could change the thermal degradation process of the SR matrix and increase its char yield, which would be beneficial to the formation of a stable char layer. 

Overall, the ability of the hollow microspheres to improve the ablation resistance of the SR composites is as follows: AMHMs > PHMs > GHM, which is the opposite of the heat resistance of the hollow microspheres. It can be concluded that the heat resistance of the filler is not the decisive factor for the evaluation of the ablation performance of the SR composites, but the heat dissipation capacity and the influence on the structure of the char layer in the ablation process are also important.

### 3.6. Thermal Insulation Performance

**Figure 7:**
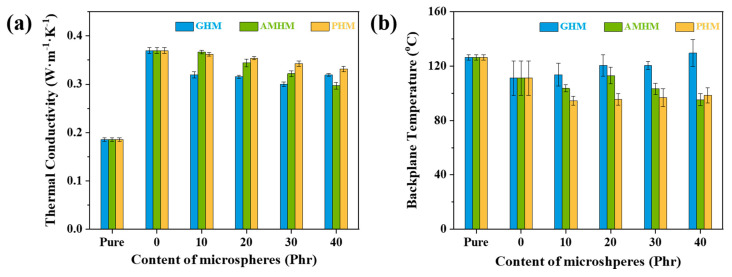
(**a**) The thermal conductivity and (**b**) the backplane temperature of the silicone rubber composites containing three types of hollow microspheres with different loading.

The thermal insulation performance of the SR composites was evaluated by the thermal conductivity and the maximum backplane temperature during ablation. Backplane temperature is a very important measurement for estimating the insulation performance of TPMs, and it is mainly determined by the thermal conductivity of the original material and the char layer, as well as the linear ablation rate. The corresponding results are shown in Table S4. As illustrated in Figure 7a, the addition of all three kinds of hollow microspheres resulted in the reduction in the thermal conductivity of the SR composites. In general, the contribution to the reduction in the thermal conductivity of the SR composites of the three types of hollow microspheres is as follows: S_N_GHMs > S_N_AMHMs > S_N_PHMs. The backplane temperature results for the S_N_GHMs, S_N_PHMs, and S_N_AMHMs are shown in Figure 7b. The backplane temperature of the S_N_GHMs gradually increased with the increasing GHM content because the high linear ablation rate of the S_N_GHMs led to the short distance from the pyrolysis layer to the backplane of the material and the heat loss in this region is limited. On the contrary, the backplane temperature of the S_N_PHM composites decreased with the incorporation of the PHMs, but it showed an insignificant change with further increasing PHM content. This insensitivity to the PHM content is due to the balancing effect between the increasing linear ablation rate and the decreasing thermal conductivity of the SNPHMs with the increasing PHM content. [40]. The addition of the AMHMs also decreased the backplane temperature of the S_N_AMHMs because of the lower linear ablation rate and the thermal conductivity. In conclusion, the backplane temperatures of the three types of hollow microsphere composites are as follows: S_N_GHMs > S_N_AMHMs > S_N_PHMs.

### 3.7. Microscopic Morphology

**Figure 8:**
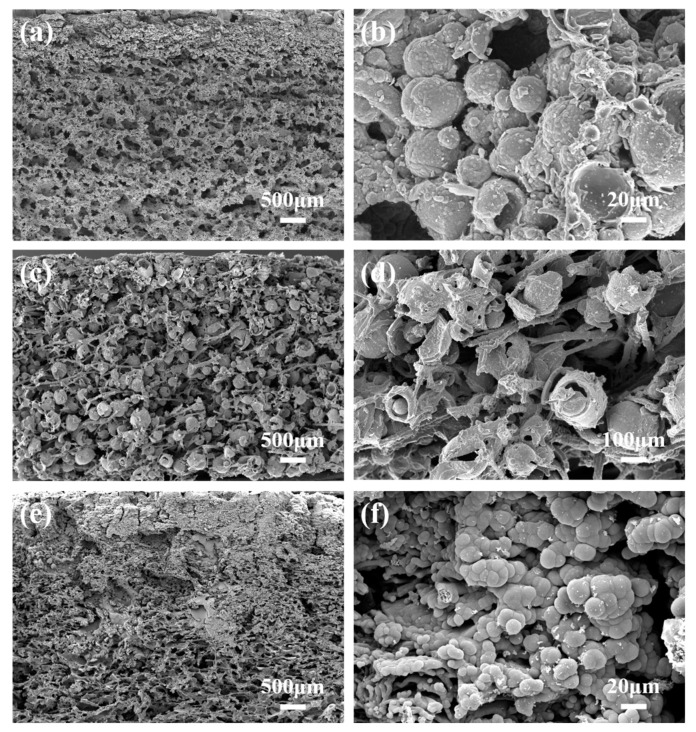
SEM images of transection morphology of the char layer for the silicone rubber composites with three hollow microspheres after ablation: (**a**,**b**) S_N_GHMs, (**c**,**d**) S_N_AMHMs, and (**e**,**f**) S_N_PHMs.

Figure 8 shows the microscopic morphology of the char layer for the S_30_GHM, the S_30_PHM, and the S_30_AMHM after ablation. As shown in Figure 8a,b, the char layer of the S_30_GHM is mainly composed of fused GHMs, but few carbonized PBO fibers were observed. Under a high temperature, the addition of the hydroxyl groups on the surface of the GHMs could have catalyzed the unzipping degradation of the SR matrix, and the high addition of GHMs may have prevented the combination of free siloxane chain radicals, inhibiting the formation of the three-dimensional Si-C-O network. In addition, the enhanced thermal conductivity of the char layer caused by melted or softened GHMs promoted the thermal degradation of PBO fibers; thus, the carbonized fibers were refined. As shown in Figure 8c,d, in the char layer of the S_30_PHM, the carbonized PHMs maintained their original microsphere structure, and the carbonized PBO fibers interwove with each other, which attributed to the strengthening of the char layer. As can be seen from the high magnification image in Figure 8d, although there was some damage, the carbonized PHM shells still remained dense, which led to the increase in the char residue and the ablation rate. As shown in Figure 8e, the char layer of the S_30_AMHMs has a clear gradient hierarchy in a longitudinal direction, with the dense upper layer and the porous lower layer showing interweaving fibers. A large amount of the char residue can be found in Figure 8f, as consistent with the TGA results, and this indicates that the addition of the AMHM is beneficial to the formation of dense char layers and the enhancement of the ablation resistance.

## 4. Conclusions

In this study, different kinds of hollow microspheres were introduced into the SR matrix to improve its heat and ablation resistance properties. The TG results showed that the introduction of the hollow microspheres of the GHMs, PHMs, and AMHMs could increase the char residue of the SR matrix and delay the T_max_ of the SR composites. The R_800_ of the SR composites for the S_30_GHMs, S_30_PHMs, and S_30_AMHMs were increased from 10.11%, in the control samples (S0), to 21.70%, 18.31%, and 20.83%, respectively. The comparison of the R_800_ and the R_T800_ revealed that the GHMs and the PHMs could increase the char residue of the SR composites due to their own higher residue mass at a high temperature, whereas the AMHMs could significantly increase the char yield by altering the thermal degradation process of the SR. The ablation results demonstrated that the addition of the GHMs and the PHMs both deteriorated the ablation resistance of the SR composites, while the addition of the AMHMs did just the opposite. The R_L_ of the S_20_AMHM was decreased by 73.03% and 18.73%, respectively, in comparison to the S_0_. The SEM images of the char layer revealed that the presence of the AMHMs with thermoplastic shells tended to promote the formation of a dense char layer of SR composites during ablation. The primary reason for the enhancement of the ablation performance of the SR composites was not the heat resistance of the hollow microspheres themselves, but their ability to dissipate heat during ablation and their impact on the structure of the char layer. This work could provide a potential method for the development of ablation resistance materials as applied to thermal protection systems. 

## Figures and Tables

**Table 1:** Compositions of the different formulations.

Samples	Ingredients in Phr							
	E-LSR	SiO_2_	PBO	GHM	PHM	AMHM	KH550	DBTDL
Pure	100	0	0	0	0	0	6	0.8
S0	100	5	6	/	/	/	6	0.8
S10/20/30/40GHM	100	5	6	10/20/ 30/40	/	/	6	0.8
S10/20/30/40PHM	100	5	6	/	10/20/ 30/40	/	6	0.8
S10/20/30/40AMHM	100	5	6	/	/	10/20/ 30/40	6	0.8

**Table 2:** The DSC results of S0, S_30_GHM, S_30_PHM, and S_30_AMHM.

Samples	T_m_ (°C)
S0	−44.51
S30GHM	−44.06
S30PHM	−43.60
S30AMHM	−43.98

## Supplementary Materials

The following supporting information can be downloaded at: https://www.mdpi.com/article/10.3390/polym14183846/s1, Table S1: The thermal gravimetric analysis data of hollow microspheres in N_2_ atmosphere. Table S2: The thermal gravimetric analysis data of composites with different content of hollow microspheres in N_2_ atmosphere. Table S3: Compositions of the different formulations. Table S4: R_L_ of composites with different content of hollow microspheres. Table S5: Thermal conductivity and backplane temperature of composites with different content of hollow microspheres.
