# Supplementary material for: Improving the Heat and Ablation Resistance of Silicone Rubber Composites by Incorporating Hollow Microspheres
Source: Polymers (Basel). 2022 Sep 14;14(18):3846. doi: 10.3390/polym14183846 (PMC9500970; doi:10.3390/polym14183846)
Supplement: Supplementary file 1 [file polymers-14-03846-s001.zip › polymers-1886586-supplementary.pdf]

Supporting Information

Heat and ablation resistance improvement of silicone rubber by incorporation of hollow microspheres

Jinfeng Tian, Liwei Yan*, Hao Zhang, Shengtai Zhou, Shuang Xia, Huawei Zou*

(The State Key Laboratory of Polymer Materials Engineering, Polymer Research Institute of Sichuan University, Chengdu 610065, China)

* Corresponding author. Tel: +86-28-85408288; Fax: +86-28-85402465.
E-mail address: liweiyan68@163.com (Liwei Yan); hwzou@163.com (Huawei Zou)

Table. S1 The thermal gravimetric analysis data of hollow microspheres in N_2 atmosphere.

Samples	T_{onset}(°C)	T_{max}(°C)	R_{800}(%)
GHM	/	/	102.14
HPM	242.7	242.1 / 445.7 / 527.7	58.74
AMHM	298.1	333.4	21.25

Table. S2 The thermal gravimetric analysis data of composites with different content of hollow microspheres in N_2 atmosphere.

Samples	T_{onset}(°C)	T_{max}(°C)	R_{800}(%)	R_{T800}(%)
Pure	363.4	402.1/448.5	7.01	-
S0	341.7	433.4	10.11	10.11
S10GHM	299.4	455.2	11.20	17.48
S20GHM	296.9	459.0	17.33	23.73
S30GHM	292.3	460.2	21.70	29.10
S40GHM	277.0	463.7	24.63	33.77
S10HPM	318.4	521.6	13.15	14.10
S20HPM	319.0	516.2	15.51	17.48
S30HPM	306.5	513.3	18.31	20.38
S40HPM	300.9	499.7	20.59	22.91
S10AMHM	269.9	453.0/581.5/666.6	13.90	11.02
S20AMHM	272.3	366.1/477.2/586.1/671.4	20.22	11.80
S30AMHM	280.6	352.5/477.2/585.3/676.4	20.83	12.46
S40AMHM	279.3	349.7/462.3/582.6/666.0	16.23	13.04

Table. S3 Compositions of the different formulations.

Samples	Ingredients in wt%				
	SiO_2	PBO	GHM	PHM	AMHM
Pure	-	-	-	-	-
S0	4.24	5.09	-	-	-
S10/20/30/40 GHM	3.91/3.63/ 3.38/3.17	4.69/4.35/ 4.06/3.80	7.82/14.51/ 20.30/25.35	-	-
S10/20/30/40 PHM	3.91/3.63/ 3.38/3.17	4.69/4.35/ 4.06/3.80	-	7.82/14.51/ 20.30/25.35	-
S10/20/30/40 AMHM	3.91/3.63/ 3.38/3.17	4.69/4.35/ 4.06/3.80	-	-	7.82/14.51/ 20.30/25.35

Table. S4 R_L of composites with different content of hollow microspheres

Samples	R_L (mm/s)
Pure	0.2415
S0	0.0803
S10/20/30/40GHM	0.0842/0.0955/0.0866/0.0899
S10/20/30/40PHM	0.0756/0.0804/0.0830/0.0865
S10/20/30/40AMHM	0.0755/0.0649/0.0730/0.0695

Table. S5 Thermal conductivity and backplane temperature of composites with different content of hollow microspheres

Samples	Thermal Conductivity ($W·m^{-1}·K^{-1}$)	Backplane Temperature (℃)
Pure	0.1855	126.29
S0	0.3693	111.09
S10/20/30/40GHM	0.3189/0.3151/0.2997/0.3189	113.48/120.45/120.45/129.37
S10/20/30/40PHM	0.3616/0.3535/0.3420/0.3316	94.52/95.34/96.74/98.28
S10/20/30/40AMHM	0.3663/0.3432/0.3216/0.2963	103.21/112.94/103.21/95.20
